# Rapid Biocompatibility Analysis of Materials via *In Vivo* Fluorescence Imaging of Mouse Models

**DOI:** 10.1371/journal.pone.0010032

**Published:** 2010-04-06

**Authors:** Kaitlin M. Bratlie, Tram T. Dang, Stephen Lyle, Matthias Nahrendorf, Ralph Weissleder, Robert Langer, Daniel G. Anderson

**Affiliations:** 1 Department of Chemical Engineering, Massachusetts Institute of Technology, Cambridge, Massachusetts, United States of America; 2 Department of Anesthesiology, Children's Hospital Boston, Boston, Massachusetts, United States of America; 3 Department of Cancer Biology, University of Massachusetts Medical School, Worcester, Massachusetts, United States of America; 4 Center for Systems Biology, Massachusetts General Hospital, Boston, Massachusetts, United States of America; 5 David H. Koch Institute for Integrative Cancer Research, Massachusetts Institute of Technology, Cambridge, Massachusetts, United States of America; Universidade do Porto, Portugal

## Abstract

**Background:**

Many materials are unsuitable for medical use because of poor biocompatibility. Recently, advances in the high throughput synthesis of biomaterials has significantly increased the number of potential biomaterials, however current biocompatibility analysis methods are slow and require histological analysis.

**Methodology/Principal Findings:**

Here we develop rapid, non-invasive methods for *in vivo* quantification of the inflammatory response to implanted biomaterials. Materials were placed subcutaneously in an array format and monitored for host responses as per ISO 10993-6: 2001. Host cell activity in response to these materials was imaged kinetically, *in vivo* using fluorescent whole animal imaging. Data captured using whole animal imaging displayed similar temporal trends in cellular recruitment of phagocytes to the biomaterials compared to histological analysis.

**Conclusions/Significance:**

Histological analysis similarity validates this technique as a novel, rapid approach for screening biocompatibility of implanted materials. Through this technique there exists the possibility to rapidly screen large libraries of polymers in vivo.

## Introduction

To our knowledge, there are no methods for *in vivo* visualization of biocompatibility or inflammatory responses to implanted biomaterials. Traditionally, biocompatibility is determined via histology. Histology allows for the determination of cell type and number near the implant, including those belonging to the immune system. However, histology is an endpoint measurement, allowing examination of only one time point per animal. Fluorescence imaging represents a set of powerful techniques that have traditionally been employed as a method for examining tumor models [1], [2], [3], [4], [5], [6], along with inflammation resulting from arthritis [7], [8], pulmonary inflammation [9], [10], and transplant rejection models [11].

When a biomaterial is implanted, the healing response is initiated by monocytes and neutrophils, followed by propagation of fibroblasts and vascular endothelial cells [12]. Infiltration of inflammatory cells can lead to such complications as: bio-instability of glucose sensors [13]; overgrowth of encapsulated pancreatic islets for diabetes therapy causing ischemia and, eventually, necrosis of the islets [14]; and constrictive fibrosis following silicone implants in mammary augmentation [15]. Granulation tissue will then be formed and may appear as early as 3 to 5 days following implantation [12]. In general, granulation tissue will ultimately form a fibrous capsule surrounding the implant [12].

Immunological responses are dynamic processes and, as such, cell type and population at the implant site change during the healing process [16]. The sequence of local events following implantation is generally regarded as the tissue response continuum in which each individual event leads to the subsequent: injury progresses to acute inflammation, which proceeds to chronic inflammation, followed by granulation tissue formation, foreign body reaction and fibrous encapsulation [16], [17]. The presence of eosinophils and polymorphonuclear (PMN) cells typify acute inflammatory responses while macrophages and fibroblasts signify the chronic form [18]. Neutrophils, together with monocytes and macrophages, release cathepsins during the process of degranulation [19], [20]. Cathepsins are proteolytic enzymes responsible for digesting foreign material [12].

Here, we describe the first methods for examining biomaterial biocompatibility in vivo, using fluorescence reflectance screening. The novelty of this technique lies in its ability to repeatedly analyze foreign body responses in the same animal. The Macrophage recruitment and protease enzyme activity, both of which serve as markers of biocompatibility, were monitored in vivo, in real-time. We believe the methods developed here provide the first rapid techniques for parallel determination of biomaterial biocompatibility *in vivo* in a non-invasive manner.

## Methods

### Molar Absorptivity

The absorbance of the two fluorophores, ProSense-680 and F4/80 pan macrophage monoclonal antibody conjugated to FITC, were monitored using UV/Vis absorbance spectroscopy over the 200 to 800 nm range. Solutions were diluted in 0.9% w/v NaCl and housed in 1 cm path-length quartz cuvettes. Absorbances were measured on a Cary 100 Bio UV/Vis Spectrophotometer.

### Ethics Statement

The research protocol was approved by the local animal ethics committees at Massachusetts Institute of Technology (Committee on Animal Care) and Children's Hospital Boston (Institutional Animal Care and Use Committee) prior to initiation of the study.

### Animals

8–12 week old male SKH1 mice were obtained from Charles River Laboratories (Wilmington, MA). The mice were maintained at the animal facilities of Massachusetts Institute of Technology, accredited by the American Association of Laboratory Animal care, and were housed under standard conditions with a 12-hour light/dark cycle. Both water and food were provided *ad libitum*.

### Injections

Injections were performed in accordance with ISO 10993-6: 2001. Prior to injection all materials were sterilized. Saline was sterilized via 0.22 µm filtration; alginate was autoclaved for 20 min. at 121°C; and polystyrene particles were washed in 70% ethanol and re-suspended in sterile saline. The mice were anesthetized via isoflurane inhalation at a concentration of 1–4% isoflurane/balance O_2_ to minimize movement. Their backs were scrubbed with 70% isopropyl alcohol and the animals were injected with saline, 2%-w/v alginate (Protanal LF 10/60, FMC BioPolymer, Newark, DE, having high guluronic acid composition (65–75%), mean molecular weight of 180kDa), or 10%-w/v polystyrene beads (3.0 µm, Sigma Aldrich, St. Louis, MO) in an array format on the mouse's back. Eight injections were made in each mouse in a random fashion to establish position-dependent inflammatory responses. Injection volumes ranged from 30–100 µl. All experiments were conducted in quadruplicate for each imaging time-point. In addition, a set four mice were imaged at every time-point and sacrificed at the 28 day time-point.

### Imaging

The following two imaging agents were co-injected into the tail vein 24 hours before *in vivo* fluorescence imaging: ProSense-680 (VisEn Medical, Woburn, MA, excitation wavelength 680±10 nm, emission 700±10 nm) [4] for imaging cathepsin activity, 2 nmol in 150 µl sterile PBS, and FITC-mAb F4/80 (Abcam, Cambridge, MA, excitation wavelength 495 nm, emission 521 nm) for imaging macrophage recruitment, 5 µg in 100 µl sterile PBS.

*In vivo* fluorescence imaging was performed with an IVIS-Spectrum measurement system (Xenogen, Hopkinton, MA). The animals were maintained under inhaled anesthesia using 1–4% isoflurane in 100% oxygen at a flow rate of 2.5 L/min. A binning of 8×8 and a field of view of 13.1 cm were used for imaging. Exposure time and f/stop – the relative size of the opening of the aperture - were optimized for each acquired image. Data were acquired and analyzed using the manufacturer's proprietary Living Image 3.1 software. All images are presented in fluorescence efficiency which is defined as the ratio of the collected fluorescent intensity to an internal standard of incident intensity at the selected imaging configuration. Regions of interest (ROIs) were determined around the site of injection. ROI signal intensities were calculated in fluorescent efficiency. Images were obtained 1, 3, 7, 14, 21, and 28 days post-injection with four replicates imaged at each time point. A separate set of four replicates were imaged at all six time points.

### Histology

Histology evaluated the severity of inflammation resulting from the injected biomaterials. Mice were euthanized via CO_2_ asphyxiation and the injected biomaterial and surrounding tissue were excised. The tissues were then fixed in 10% formalin, embedded in paraffin, cut into 5 µm sections, and stained using hematoxylin and eosin (H&E) for histological analysis by a board certified pathologist. Fibrosis was rated on a scale where a zero involved no fibrosis, a one indicated partial coverage with one to two layers of fibrosis, a two is designated a thicker fibrotic layer that nearly covered the implant, and a three denoted concentric fibrotic coverage of the polymer. Both polymorphonuclear (PMN) cells and macrophages were rated on a scale where no observed cells were indicated with a zero, scattered cells scored a one, numerous cells clustering on the sides of the polymer scored a two, and numerous cells surrounding the material resulted in a three.

### Statistical Analysis

The values of the histologic scores and the ROIs were averaged and expressed as the mean ± standard error of the mean. Comparisons of values were performed by the Student's unpaired two-tailed *t*-test. *P* values less than 0.05 were considered significant.

## Results

### Linearity of Fluorescence Response to Dose Concentration

Prior to quantifying responses *in vivo*, the linearity of the *in vitro* fluorescence response to concentration of dye was assessed, facilitated by the stationary superficially implanted target Fluorescence intensity 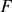 is proportional to the intensity of the excitation beam that is absorbed by the system. That is,
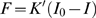
(1)


Where 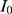 is the intensity of the incident excitation beam and 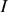 is the detected fluorescence intensity after traversing a length 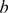 of the medium - in this case - the tissue of the animal. The constant 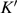 depends upon the quantum efficiency of the fluorescence process. In order to relate 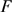 to the concentration 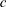 of the fluorescing species, Beer's law can be written in the form:
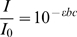
(2)


Where 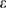 is the molar absorptivity of the fluorescing molecules and 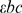 is the absorbance. Inserting Beer's law into equation 1, we obtain:
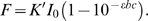
(3)


Which can be approximated for absorbances less than 0.05 to:
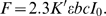
(4)


Assuming that 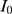 is constant, the fluorescence intensity is linearly proportional to concentration at low absorbances. The molar absorptivities were determined by UV-visible absorbance to be 2.90±0.04×10^6^ M^−1^ cm^−1^ and 2.30±0.06×10^5^ M^−1^ cm^−1^ for ProSense-680 and FITC mAb-F4/80, respectively. With an *in vivo* penetration depth for visible light of ∼5 mm in reflectance mode [1], the onset of nonlinear relations between fluorescence and concentration would present at doses 5 and 2.5 times larger than those injected for FITC mAb-F4/80 and ProSense-680, respectively, indicating the ability for relative quantitative analysis.

### *In Vivo* Imaging of Cathepsin Activity and Macrophages

**Figure 1:**
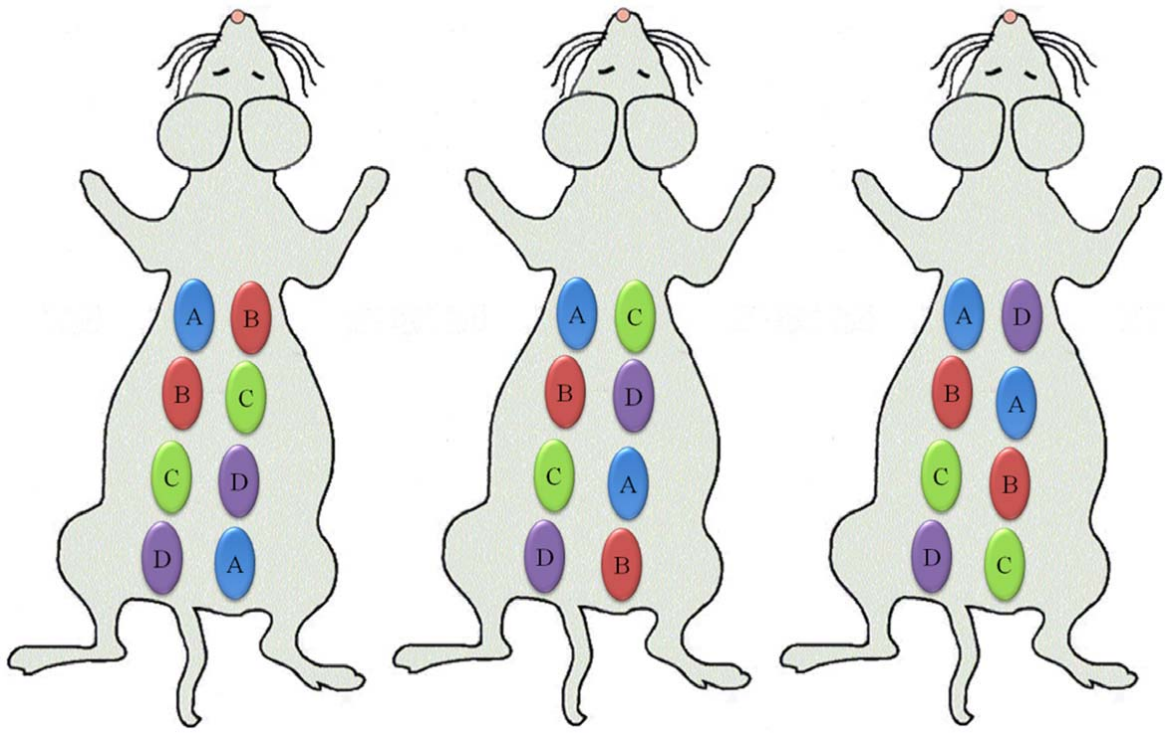
Subcutaneous Injection arrays. Three array formats used for injecting saline and polymers subcutaneously in mice where A is 30 µl, B is 50 µl, C is 70 µl, and D is 100 µl.

**Figure 2:**
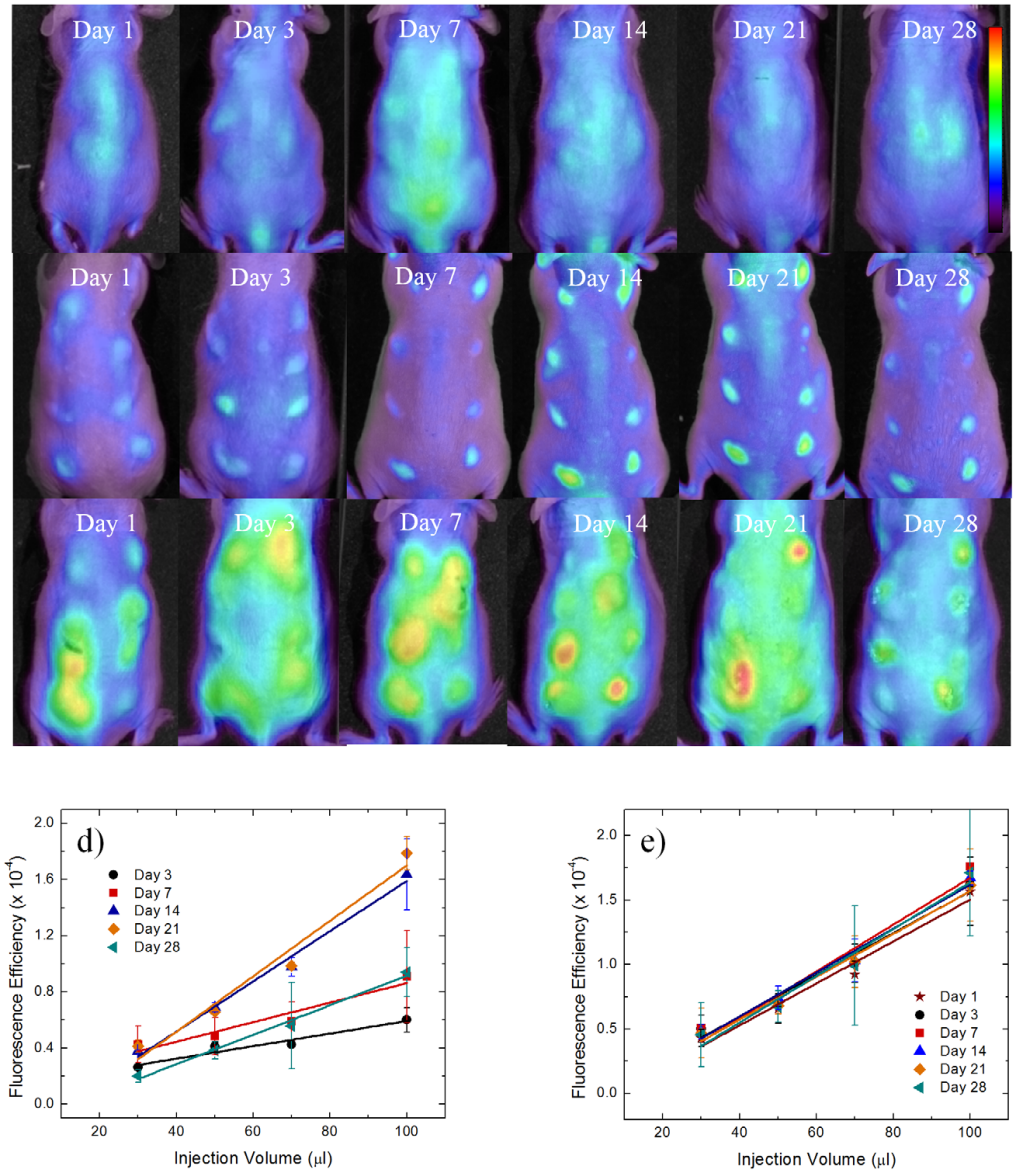
Time evolution of cathepsin activity in response to injected materials fluorescently imaged. *In vivo* fluorescence imaging using ProSense 680 for cathepsin activity at various time points for a) saline, b) polystyrene, and c) alginate. The scale bar ranges 0–6×10^−4^ in fluorescence efficiency. The quantified fluorescence efficiencies of cathepsin activities are shown for d) polystyrene and e) alginate as the mean with standard deviation. Symbols represent data points and lines represent linear regressions.

**Figure 3:**
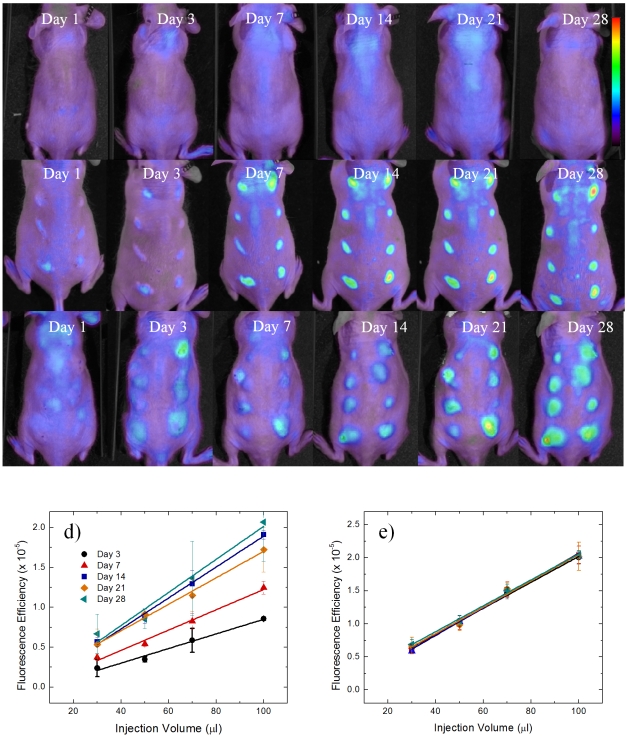
Time evolution of macrophage response to injected materials fluorescently imaged. *In vivo* fluorescence imaging of F4/80 pan macrophage antibody at various time points for a) saline, b) polystyrene, and c) alginate. The scale bar ranges 0–1.5×10^−4^ in fluorescence efficiency. The quantified fluorescence efficiency of F4/80 pan macrophage responses are shown for d) polystyrene and e) alginate as the mean with standard deviation. Symbols represent data points and lines represent linear regressions.

Mice were injected with alginate, polystyrene, or saline in an array format (figure 1) in volumes of 30, 50, 70, and 100 µl. Alginate is a bio-inert material used in a variety of biomedical applications including encapsulation of insulin producing islets for diabetes therapy [21], [22], [23], [24], wound healing [25], [26], implants for cardiac remodeling following infarction [27], [28]. In contrast, Polystyrene exhibits high cellular adhesive properties, induces a strong inflammatory response and was chosen as a positive control. Polystyrene particles below 10 µm activate macrophages and are easily phagocytosed [29], allowing them to serve as positive controls. Saline serves as a negative control to assess the background fluorescence level and aid in determination of the detection limit. After injection, the mice were imaged at prescribed time points for cathepsin activity and macrophages as shown in figures 2a–c and 3a–c. Fluorescent regions of interest (ROIs) were quantified for each image and are presented in figures 2d,e and 3d,e for cathepsin activity and macrophages recruitment.

Qualitatively, for saline, the cathepsin activity and macrophage fluorescent signal appear to be very low with the exception of cathepsin activity on day 7. For polystyrene, cathepsin activity follows very similar trends wherein protease activity is detected on day one, peaks at three weeks, and begins to decline at four weeks. Recruitment of immune cells to alginate displays a different trend than polystyrene in which cathepsin activity remains constant from the first day to the fourth week. Macrophage recruitment for polystyrene reached a plateau at day seven. Alginate arrived at this plateau earlier, at the third day.

Quantitative performance criteria of methods are necessary in determining whether this technique is suitable in analyzing inflammatory responses. Detection limits are defined as the blank plus three times the standard deviation of the blank and limit of quantitation (LOQ) is ten times the standard deviation of the blank. For macrophage detection, the detection limit is a fluorescence efficiency of 9.4×10^−7^ and the LOQ is a fluorescence efficiency of 2.3×10^−6^. ProSense-680 has a fluorescence efficiency detection limit of 1.1×10^−5^ and the fluorescence efficiency LOQ is 1.8×10^−5^. Quantification of fluorescence efficiency of both cathepsin activity and macrophage recruitment is above the LOQ as shown in figures 2 and 3. Cathepsin activity on the first day after injection of polystyrene was not above the LOQ and therefore not included in figure 2d. Macrophage recruitment on day one for alginate and polystyrene were also below the LOQ and not included in figure 3d and e.

### Histology

**Figure 4:**
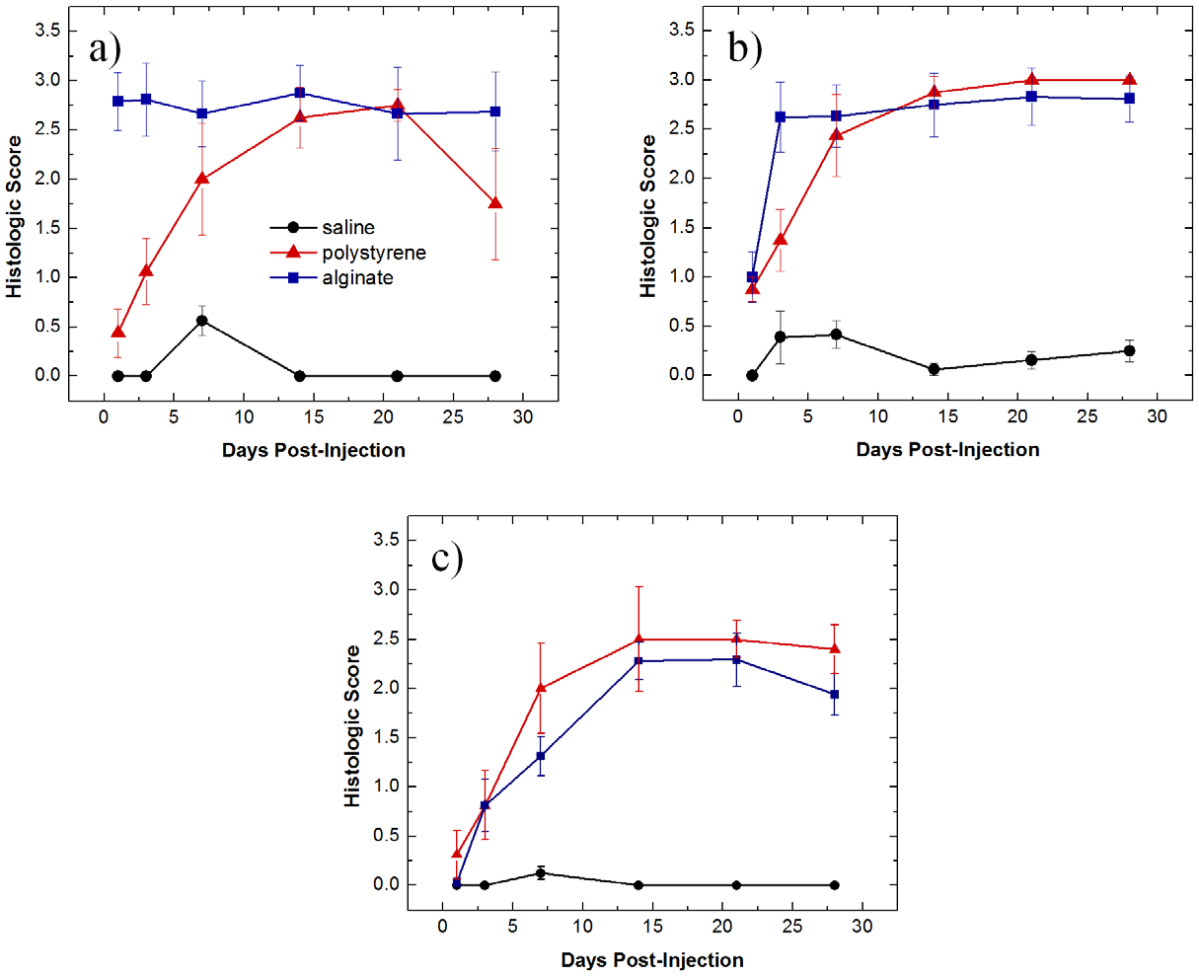
Histological scores of materials subcutaneously injected. Histological scores of a) neutrophils, b) macrophages, and c) fibrosis determined for tissue excised at various time points with injections of saline, polystyrene, and alginate. Values shown are means with standard deviations.

**Figure 5:**
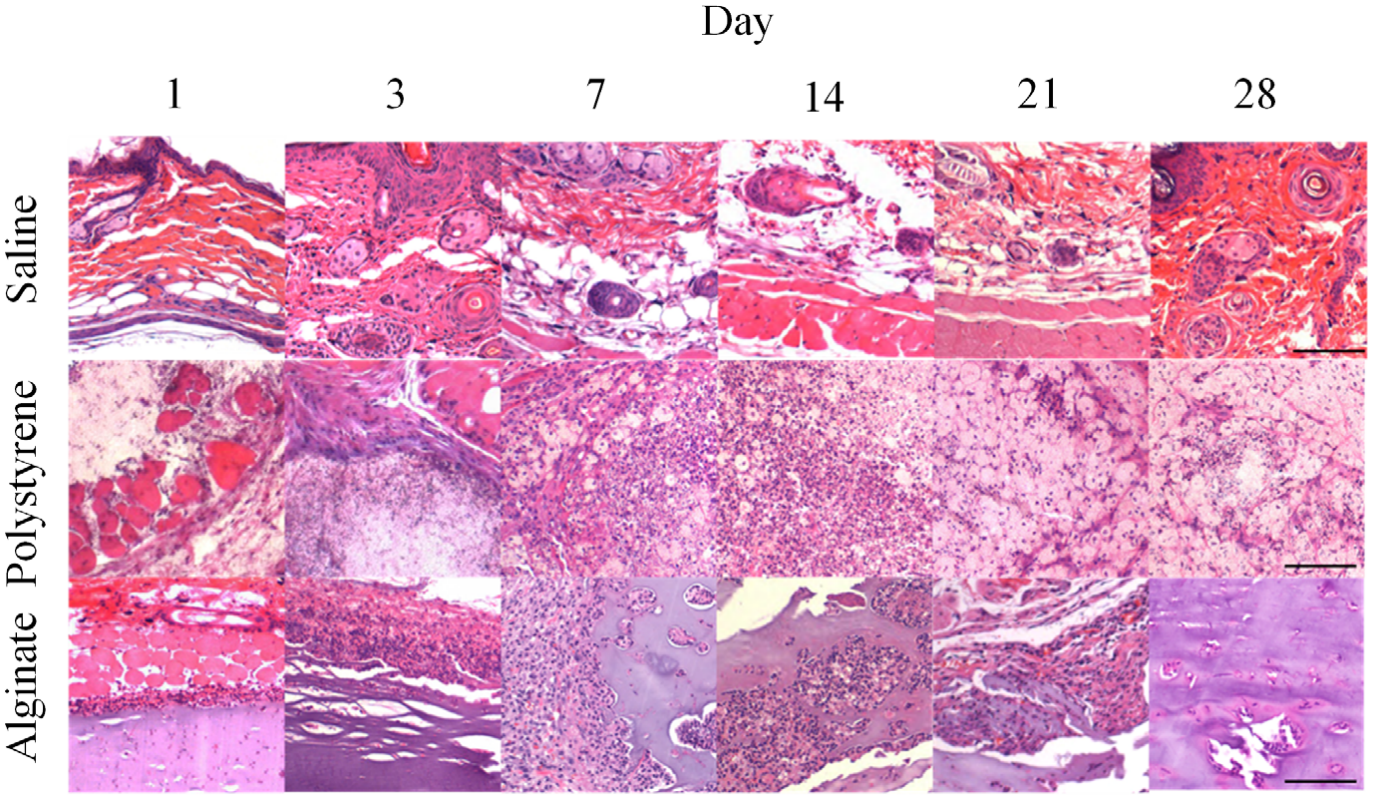
H&E staining of representative sections subcutaneously injected. Representative sections stained with H&E are shown for saline, polystyrene, and alginate at various time points (1, 3, 7, 14, 21, and 28 days post-injection). (Magnification 20×, scale bar = 100 µm).

Validation of the *in vivo* imaging technique for biocompatibility described required histologic analysis subsequent to each imaging time point. Several inflammation markers were quantified: PMNs, macrophages, and fibrosis. PMNs and macrophages were scored on the basis of zero being normal cell populations, one being scattered cells, two being numerous cells mostly populating the sides of the polymer, and three being the most severe where numerous cells surrounded the material. Quantified scores and representative images are shown in figures 4 and 5, respectively. Minimal PMNs are seen infiltrating the injection site for saline whereas for alginate neutrophils completely surround the injection site from day one to day 28. For polystyrene, neutrophils are present the first day following injection, reaching a maximum population at day 21 and subsequently decreasing.

Macrophage recruitment for the saline injections is very low (Fig. 4b). Slightly elevated levels of macrophages on days three and seven likely result from trauma of the injury, not the biocompatibility of saline, and agrees with previous results [30]. A more pronounced reaction occurs in response to polystyrene and alginate. Polystyrene reaches a plateau seven days post-injection, while alginate levels out at day three. The size of the polystyrene particles (3.0 µm) lends to being easily phagocytosed, which can be seen in figure 5.

Fibrosis of the implants was also analyzed histologically in which a score of zero denotes no fibrosis, one signifies partial coverage with one to two layers of fibroblasts, two indicates a thicker layer nearly covering the implant, and three represents concentric fibrotic coverage of the polymer. As seen in figure 4c, fibrosis for alginate and polystyrene gradually increases reaching a maximum at fourteen days. This observation is in line with previous findings [31] in which wound dressings of calcium alginate were grafted in porcine models and found fibrosis to reach a maximum at 14 days. The slight decrease in fibrosis scoring at day 28 might result from myofibroblasts contracting the wound as part of the healing process [32].

## Discussion

Chemical signals responsible for invoking a response toward implanted biomaterials may include proteins from invading bacteria, clotting system peptides, complement products, and cytokines that have been released by macrophages located in the tissue near the implantation site [33]. Another group of chemical attractants are chemokines which recruit neutrophils and monocytes from the blood [34]. Macrophages derive from monocytes [35]. Macrophages and monocytes can phagocytose cellular debris and pathogens, and stimulate lymphocytes and other immune cells to respond to the pathogen.

Typically for acute inflammation, neutrophil recruitment peaks 1–2 days after implantation and gradually resolves after 7–10 days followed by macrophage migration at 1–2 days after injury [36]. Fibroblasts typically infiltrate at 2–3 days reaching a maximum population at 3–4 days [36]. Both macrophages and fibroblasts disperse after 5–9 days [36]. Chronic inflammation also begins with recruitment of neutrophils [37]. Additionally, protease levels are reported to be higher in chronic wounds [38]. Fibroblasts and macrophages become numerous one to two weeks after injury and diminish at six weeks [36], [39]. Histologic analysis and *in vivo* fluorescence imaging showed very similar trends in macrophage recruitment and also in comparing cathepsin activity derived from *in vivo* imaging to neutrophils evaluated via histology, suggesting that a significant portion of the protease secreted derives from neutrophils. Cathepsin may also derive from macrophages [40]. Christen *et al.* [11] have shown that ∼75% of the prosense signal is macrophage derived in transplant rejection. Early markers of inflammation – macrophages and cathepsin activity – have been chosen to assess biocompatibility of various polymers.

Traditionally, the local pathological effect of a material on living tissue that is placed into an implant site is evaluated at both the gross level and the microscopic level. Various biological parameters such as cellular responses and histopathological changes are evaluated via ex vivo histology [17]. The throughput of histology is typically on the order of days to several weeks and involves steps such as fixation, embedding, processing, and staining. *In vivo* fluorescent images can be acquired in minutes and only require anesthetization of the subject, thus greatly reducing the time required to screen libraries of compounds. Recently, Sabaliauskas *et al.* [41] have made advances in improving the throughput of histology by automating and digitizing data acquisition. Gersner *et al.* [42] have developed laser scanning cytometry methods to quantify histological specimens, increasing the throughput of analysis. Specimen preparation still remains a costly, labor-intensive bottleneck in histology and, thus, in biocompatibility screening.

Aside from quantitative detection limit and LOQ, comparison of the dynamic ranges between histology and *in vivo* imaging is also necessary in determining the abilities of fluorescence imaging in assessing immune responses. In comparing histologic scores of polystyrene with fluorescence imaging for cathepsin activity (neutrophils), scores greater than 0.5 are above the detection limit, meaning that the injection sites are distinguishable from the background autofluorescence of the mouse. Histologic scores above 1 appear to correlate to fluorescence efficiencies above the LOQ for cathepsin activity (neutrophils). Comparing fluorescence imaging to histologic scores for macrophages leads to the conclusion that the detection limit and LOQ obtained for *in vivo* imaging corresponds to a histologic score of 1.5, indicating the possibility for false negatives in detecting macrophage infiltration and the necessity for histologic analysis. However, the use of amplification mechanisms such as use of fluorescent nanoparticles avidly taken up by macrophages [4] will likely enhance sensitivity drastically. Although this technique is semi-quantitative owing to the poor depth penetration of visible light [1], in conjunction with histology it possesses the ability to transform the rapidity with which libraries of novel materials are assessed for biocompatibility.

The methods developed here provide for rapid, in vivo analysis of several different materials simultaneously, thereby allowing for rapid, kinetic analysis of the foreign body response to a number of biomaterials, as well as eliminating labor intensive tissue processing steps typically necessary for histology. We anticipate that *in vivo* fluorescence imaging may therefore help address bottlenecks in analyzing biocompatibility of polymers and aid in understanding foreign body responses to biomaterials. *In vivo* fluorescence imaging also holds the advantage of monitoring temporal immune cell changes, thus eliminating mouse-to-mouse variations present when making a static histologic assessment.
